# Magnesium Links Starvation-Mediated Antibiotic Persistence to ATP

**DOI:** 10.1128/mSphere.00862-19

**Published:** 2020-01-08

**Authors:** Tao Xu, Xuyang Wang, Lu Meng, Mengqi Zhu, Jing Wu, Yuanyuan Xu, Ying Zhang, Wenhong Zhang

**Affiliations:** a Department of Infectious Diseases, National Clinical Research Center for Aging and Medicine, Huashan Hospital, State Key Laboratory of Genetic Engineering, School of Life Science, Key Laboratory of Medical Molecular Virology (MOE/MOH) and Institutes of Biomedical Sciences, Shanghai Medical College, Fudan University, Shanghai, China; b Key Laboratory of Molecular Virology and Immunology, Institut Pasteur of Shanghai, Chinese Academy of Sciences, Shanghai, China; c Department of Molecular Microbiology and Immunology, Bloomberg School of Public Health, Johns Hopkins University, Baltimore, Maryland, USA; University of Rochester

**Keywords:** ATP, *Staphylococcus aureus*, antibiotic persistence, magnesium

## Abstract

Bacterial persisters emerge and increase in numbers over time as a bacterial culture grows from log phase to stationary phase. However, the underlying basis of the inevitable tendency is unclear. In this study, we investigated the role of nutrients in starvation-mediated persister formation of Staphylococcus aureus. By screening of nutrient components, we found that starvation-induced persister formation of log-phase cultures could be reversed by addition of magnesium (Mg^2+^) but not amino acids, nucleotides, or other salts. Further, deprivation of extracellular Mg^2+^ reduced cytoplasmic ATP, inducing persistence without affecting cytoplasmic Mg^2+^ or membrane potential. Finally, we showed that Mg^2+^ reduced expression of stationary cell marker genes, *cap5A* and *arcA*. These findings indicate a connection between Mg^2+^ levels and ATP, which represents metabolic status and mediates antibiotic persistence during growth.

**IMPORTANCE** Various genes have been identified to be involved in bacterial persister formation regardless of the presence or absence of persister genes. Despite recent discoveries of the roles of ATP and membrane potential in persister formation, the key element that triggers change of ATP or membrane potential remains elusive. Our work demonstrates that Mg^2+^ instead of other ions or nutrient components is the key element for persistence by inducing a decrease of cytoplasmic ATP, which subsequently induces persister formation. In addition, we observed tight regulation of genes for Mg^2+^ transport in different growth phases in S. aureus. These findings indicate that despite being a key nutrient, Mg^2+^ also served as a key signal in persister formation during growth.

## Abstract (precis)

Various genes have been identified to be involved in bacterial persister formation regardless of the presence or absence of persister genes. Despite recent discoveries of the roles of ATP and membrane potential in persister formation, the key element that triggers change of ATP or membrane potential remains elusive. Our work demonstrates that Mg^2+^ instead of other ions or nutrient components is the key element for persistence by inducing a decrease of cytoplasmic ATP, which subsequently induces persister formation. In addition, we observed tight regulation of genes for Mg^2+^ transport in different growth phases in S. aureus. These findings indicate that despite being a key nutrient, Mg^2+^ also served as a key signal in persister formation during growth.

## OBSERVATION

Persisters, defined as a subpopulation of bacterial cells that survive lethal doses of antibiotics by entry into dormancy, play a key role in persistent bacterial infections (1, 2). Multiple genes from different physiological processes, including DNA repair, protein synthesis, toxin-antitoxin, and energy production, have been shown to affect persister formation (3–7). However, most persister genes are neither essential nor conserved for persistence among different bacteria (7). Meanwhile, the phenomenon that the number of persisters of a given culture increases as the culture ages and enters stationary phase is ubiquitous among all bacterial species (8). Therefore, the formation of bacterial persisters is a complicated process, with multiple redundant mechanisms that lead to profound changes in physiological status. Recent studies showed that starvation is a major trigger of antibiotic persistence. Starvation of Escherichia coli cells in biofilms produces higher tolerance to ofloxacin (9). Chen et al. (33) showed that poly(dC)/RmlB transduces signals of starvation to mediated persistence in Pseudomonas aeruginosa. Two expected consequences of starvation, decreases in ATP levels and membrane potential, proved to be two important aspects in persister formation (3, 10, 11). However, there are still missing links such as signal sensing and regulatory mechanisms between starvation and ATP/membrane potential-mediated persister formation.

**FIG 1:**
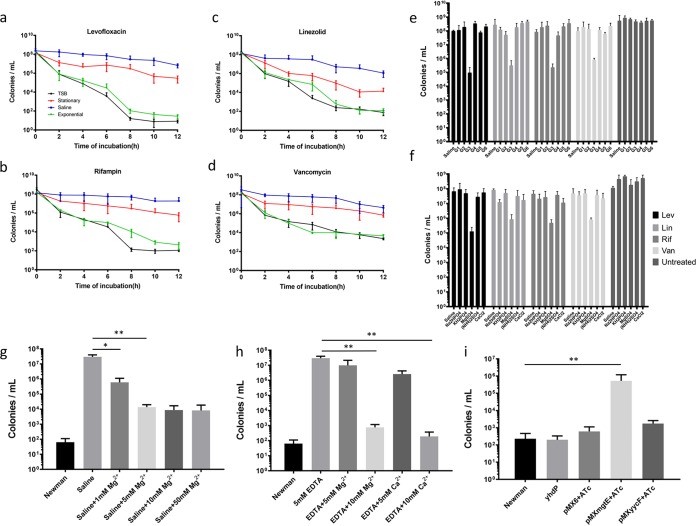
Magnesium dampens starvation-induced persistence. (a to d) Persister levels of S. aureus Newman log-phase cultures treated with saline or supernatant of stationary-phase cultures. Bacterial samples were treated with different antibiotics as described in the text for 8 h, and CFU counting was performed every 2 h. (e) Persister levels of Newman log-phase cultures treated with saline and different groups of HHWm. (f) Persister levels of Newman log-phase cultures treated with saline and different components from the major salt group of HHWm. (g) Levels of persisters against levofloxacin of Newman exponential cultures treated with saline and different concentration of Mg^2+^. (h) Levels of persisters against levofloxacin from Newman exponential cultures treated with EDTA and Mg^2+^ or Ca^2+^. (i) Levels of persisters against levofloxacin from exponential cultures of the *yhdP* mutant strain or Newman with induced asRNA against *mgtE*. Results are expressed as CFU count with comparison to untreated cultures. Data are the average results from at least two independent experiments, each with three biological replicates. Signiﬁcant differences are indicated with asterisks: *, *P* < 0.05; **, *P* < 0.01 (two-way ANOVA). Standard deviations are represented with error bars.

To study the effects of starvation on Staphylococcus aureus persister cell formation, we examined persister levels of log-phase cultures under nutrient deprivation by resuspending the bacterial cells in saline or supernatants of stationary-phase cultures. As shown in Fig. 1a to d, treatment of log-phase cultures with stationary-phase supernatants significantly induced persister formation compared to that with log-phase supernatants, and treatment with saline caused even stronger induction of persisters. To investigate the key nutrient that mediates persister formation, we used a chemically deﬁned medium (Hussain-Hastings-White modiﬁed medium [HHWm]) that supports S. aureus growth and persister formation (see Fig. S1 in the supplemental material) (12). The components of HHWm were grouped into six groups including amino acids, sugar, major salts, trace salts, nucleotides, and trace compounds (concentrations of each component are listed in Table S1). By testing the persister formation of log-phase S. aureus treated with saline or saline supplemented with different nutrient groups diluted to the same concentration as in HHWm, we found that only the major salt group reduced persistence (Fig. 1e). A further dissection with each salt component showed that it was MgSO_4_ that dampened induction of persisters (Fig. 1f). Since an extra control Na_2_SO_4_ showed no effect in reversing persister formation, it was confirmed that Mg^2+^ but not SO_4_^2−^ was the functioning factor. Indeed, addition of Mg^2+^ up to 5 mM showed the strongest inhibition of persister formation (Fig. 1g). We further showed that the effects of Mg^2+^ are applicable in different S. aureus strains, including methicillin-resistant strains USA300_FPR3757, USA500 (13), and a reference model strain, HG003 (Fig. S2) (14). Treatment with EDTA, a cation-chelating agent, significantly enhanced persister formation, which could be inhibited by Mg^2+^. Although Ca^2+^ was unable to hinder persister induction by nutrition depletion, it could free Mg^2+^ and offset the effects of EDTA on persister formation (Fig. 1h), probably by its higher affinity for EDTA than Mg^2+^ (15).

10.1128/mSphere.00862-19.1 FIG S1 Persister levels of Newman exponential cultures with medium tryptic soy broth (TSB) or HHWm. The cultures were treated with different antibiotics as described in the text for 12 h, and CFU counting was performed. Download FIG S1, TIF file, 0.4 MB. Copyright © 2020 Xu et al. 2020 Xu et al. This content is distributed under the terms of the Creative Commons Attribution 4.0 International license.

10.1128/mSphere.00862-19.2 FIG S2 Persister levels of S. aureus strains. The log-phase cultures were treated with levofloxacin as described in the text for 12 h, and CFU counting was performed. Download FIG S2, TIF file, 0.2 MB. Copyright © 2020 Xu et al. 2020 Xu et al. This content is distributed under the terms of the Creative Commons Attribution 4.0 International license.

10.1128/mSphere.00862-19.6 TABLE S1 Grouping and concentrations of HHWm components. Download Table S1, DOCX file, 0.02 MB. Copyright © 2020 Xu et al. 2020 Xu et al. This content is distributed under the terms of the Creative Commons Attribution 4.0 International license.

We then tested whether disruption of magnesium transport or efflux could affect persister formation. MgtE is a vital Mg^2+^ transporter in S. aureus Newman (16), and YhdP is the efflux pump for Mg^2+^ (17). We constructed a mutant strain of *yhdP* by homologous recombination but could not obtain a knockout mutant of *mgtE*. Instead, we used an antisense RNA (asRNA) plasmid that targets *mgtE* to address the role of *mgtE* in persister formation. Silencing of the essential *mgtE* inhibited growth, while the *yhdP* mutant showed no growth defect (Fig. S3). Silencing *mgtE* caused significant increase of persisters, while mutation of YhdP did not affect persister formation of either log- or stationary-phase cultures, As a control, asRNA against *yycF*, which is also an essential gene, did not increase persister formation (Fig. 1i).

10.1128/mSphere.00862-19.3 FIG S3 Growth curve of Newman *yhdP* mutant strain or Newman with induced asRNA against *mgtE* or *yycF*. Download FIG S3, TIF file, 0.3 MB. Copyright © 2020 Xu et al. 2020 Xu et al. This content is distributed under the terms of the Creative Commons Attribution 4.0 International license.

**FIG 2:**
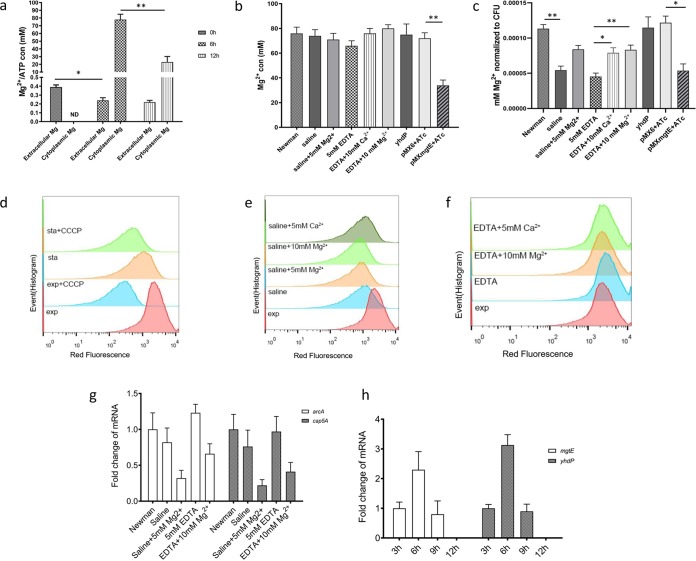
ATP but not membrane potential mediates Mg^2+^-associated persistence. (a) Quantitation of cytoplasmic and extracellular concentrations of Mg^2+^ as well as cytoplasmic ATP in growth. (b) Detection of cytoplasmic Mg^2+^ and ATP levels of Newman strain treated with saline or EDTA. (c) Antisense RNA of *mgtE* decreases cytoplasmic Mg^2+^ and ATP. (d to f) Impacts of growth phase, saline, or EDTA on membrane potential of the Newman strain. The red fluorescence that indicates membrane potential was analyzed by fluorescence-activated cell sorting. sta, stationary phase; exp, exponential phase; CCCP, carbonyl cyanide *m*-chlorophenylhydrazone. (g) Mg represses expression of *arcA* and *cap5A* in Newman exponential cultures treated with saline or EDTA, detected by qRT-PCR. (h) Expression levels of *mgtE* and *yhdP* in different growth phases. Data represent the results from three independent experiments with standard deviations represented with error bars. Signiﬁcant differences are indicated with asterisks: *, *P* < 0.05; **, *P* < 0.01 (two-way ANOVA); ND, no data.

Mg^2+^ is the most abundant multivalent cation in all living cells. Bacteria maintain a high concentration of cytoplasmic Mg^2+^, which results in a concentration gradient up to hundreds of times across the cell membrane (18). Magnesium participates in all biological pathways that produce or consume ATP (19). During entry into stationary phase, persister formation has been correlated with a decrease of cytoplasmic ATP (3, 10). However, the direct link between growth status and change of ATP levels is missing. We speculated that a change in extracellular or cytoplasmic Mg^2+^ concentration could affect persister formation and that ATP participated in this effect. First, we measured the concentrations of extracellular and cytoplasmic Mg^2+^ in log phase or stationary phase. The result is depicted in Fig. 2a, demonstrating that the Mg^2+^ in culture medium was significantly consumed from inoculation to log phase but remained at a stable concentration until stationary phase. The cytoplasmic Mg^2+^ of stationary-phase cells decreased by ∼70%, compared with that of log-phase cells, showing a similar trend as the drop in ATP from log phase to stationary phase in S. aureus (3). It is worth noting that from inoculation to log phase, the bacteria consumed one-third of the Mg^2+^ in the medium, but the concentration of Mg^2+^ in the medium was not significantly decreased during growth from log phase to stationary phase, while the cytoplasmic Mg^2+^ of bacteria was reduced by two-thirds. With an ∼5-fold increase of bacterial population from late exponential phase to stationary phase, it appears that the daughter cells went through loss of cytoplasmic magnesium during reproduction.

To study whether the drop in extracellular or cytoplasmic Mg^2+^ could cause a decrease of cytoplasmic ATP, the effects of treatment with saline or EDTA on cytoplasmic Mg or ATP were determined. While we found little influence on cytoplasmic Mg^2+^ by treatment with saline or EDTA, the ATP levels were significantly reduced. Like persistence, the effects of saline on cytoplasmic ATP could be dampened by addition of Mg^2+^, and the effects of EDTA could be dampened by addition of Mg^2+^ or Ca^2+^ (Fig. 2b and c). The concentration of cytoplasmic Mg^2+^ decreased under induction of *mgtE* asRNA but was not affected by depletion of *yhdP* (Fig. 2b). Similar effects were observed on the concentration of cytoplasmic ATP (Fig. 2c).

It is intriguing that depletion of Mg^2+^ from medium caused a rapid drop in cytoplasmic ATP without affecting the concentration of cytoplasmic Mg^2+^, indicating that magnesium serves as a signal that bacteria utilize to cope with the approaching magnesium starvation by reducing ATP as a counterstrategy. A similar strategy has been demonstrated by a series of studies in Salmonella enterica, which copes with low magnesium stress by reducing cytoplasmic ATP to allow translation by ribosomes (20). The PhoP/PhoQ two-component system (TCS) is the sensor for extracellular Mg^2+^, and the virulence protein MgtC mediates reduction of ATP production (21). However, in S. aureus the sensor of either extracellular or cytoplasmic Mg^2+^ remains unknown, and there is no close homolog of MgtC or M-box, the magnesium-sensing riboswitch RNA (22). PhoPQ is the typical extracellular Mg^2+^ sensor in Gram-negative bacteria but not in S. aureus. We investigated the roles of several TCSs (PhoPR, ArlRS, SrrAB, and GraRS) by detecting the persister level of their mutants. None of them seemed to be involved in starvation-triggered antibiotic persistence (Fig. S4).

10.1128/mSphere.00862-19.4 FIG S4 Persister levels of S. aureus strains and their knockout mutation with TCS genes. The log-phase cultures were treated with different antibiotics as described in the text for 12 h, and CFU counting was performed. Download FIG S4, TIF file, 0.2 MB. Copyright © 2020 Xu et al. 2020 Xu et al. This content is distributed under the terms of the Creative Commons Attribution 4.0 International license.

Membrane potential has been found to be responsible for persister induction by inactivation of tricarboxylic acid (TCA) genes in S. aureus (11). However, a recent study showed that extracellular magnesium suppresses membrane potential-mediated hyperpolarization and protects Bacillus subtilis from ribosome-targeting antibiotics (23). Here, in S. aureus we showed that treatment with saline significantly reduced the membrane potential of the log-phase cultures, and while addition of Mg^2+^ reduced persister cell formation, it caused a slight decrease in membrane potential of saline-treated cultures (Fig. 2d to f). Therefore, the sensitization of persisters by magnesium was not due to recovery of membrane potential.

Two reporter genes, *arcA* and *cap5A*, have been found to be activated in stationary phase and used as indicators for persister formation (24). The expression of *arcA* and *cap5A* was slightly induced by treatment with saline or EDTA but significantly repressed by addition of Mg^2+^ (Fig. 2g). We also detected the expression of *mgtE* or *yhdP*, each of which was upregulated from inoculation to log phase, while during entry to stationary phase the two genes were silenced to undetectable levels (Fig. 2h). This indicates that S. aureus shuts down magnesium transport and export after entry into stationary phase, although the nutrition in the supernatant of a stationary-phase culture is able to support a new round of bacterial growth to stationary phase (unpublished data). This might provide an explanation for our observation that addition of extra Mg^2+^ to stationary-phase cultures could not reduce persisters of stationary-phase cultures (Fig. S5). Further studies on sensors for extracellular and cytoplasmic Mg^2+^ will provide clues for unraveling the detailed mechanism of magnesium-mediated persister formation in S. aureus.

10.1128/mSphere.00862-19.5 FIG S5 Persister levels of stationary-phase cultures of S. aureus treated with additional Mg^2+^. The cultures were treated with different antibiotics as described in the text for 5 days, and CFU counting was performed. Download FIG S5, TIF file, 0.2 MB. Copyright © 2020 Xu et al. 2020 Xu et al. This content is distributed under the terms of the Creative Commons Attribution 4.0 International license.

## 

### Bacterial strains and genetic manipulations.

S. aureus Newman and USA300_FPR3757 were used, unless otherwise stated, for this study. Mutant strains of USA300 were selected from the sequence-defined transposon mutant library of S. aureus (25). The *yhdP* mutant of the Newman strain was obtained with plasmid pMX10-yhdP, which was constructed by inserting two DNA fragments beside *yhdP* into the plasmid pMX10 (26), according to the standard homologous recombination procedure (27). The antisense RNA plasmid against *mgtE* or *yycF* was constructed by inserting a DNA fragment that covered the ribosome binding site and the following ∼100 bp of the coding sequence of the target gene into pMX6 (28). Plasmid constructions were performed with E. coli strain DC10B (29). More details of strains and plasmids are listed in Table S2, and sequences of primers used in this study are listed in Table S3.

10.1128/mSphere.00862-19.7 TABLE S2 Strains and plasmids used in this study. Download Table S2, DOCX file, 0.02 MB. Copyright © 2020 Xu et al. 2020 Xu et al. This content is distributed under the terms of the Creative Commons Attribution 4.0 International license.

10.1128/mSphere.00862-19.8 TABLE S3 Primers used in this study. Download Table S3, DOCX file, 0.02 MB. Copyright © 2020 Xu et al. 2020 Xu et al. This content is distributed under the terms of the Creative Commons Attribution 4.0 International license.

### Mg^2+^ quantitation.

For cytoplasmic Mg^2+^, the bacterial cells were collected and resuspended in PBS containing 100 μg of lysostaphin (Sigma, Germany). The cells were thoroughly lysed at 37°C for 0.5 to 1 h. The Mg^2+^ concentration was detected with the QuantiChrom magnesium assay kit (Bioassay Systems, Hayward, CA), according to the recommended protocol. The final concentration was normalized to CFU and average volume of S. aureus in log phase (0.33 μm^3^) or stationary phase (0.23 μm^3^) (30).

### Persister assays.

The cultures grown to exponential phase (OD_600_ = 0.5) were centrifuged and resuspended with saline, 5 mM EDTA, or 5 or 10 mM MgSO_4_. The cultures were incubated at 37°C for 30 min and treated with 10× to 100× MIC of levofloxacin (50 μg/ml), linezolid (25 μg/ml), rifampin (1 μg/ml), or vancomycin (50 μg/ml) for 8 to 12 h. Antibiotics were removed by washing, and serial dilutions of each sample were performed with 10-μl aliquots spotted on tryptic soy agar (TSA) plates for CFU counting. Results were obtained from three biological duplicates, and the data were assessed with the *t* test.

### ATP assays.

The cytoplasmic ATP concentration was measured with the BacTiter-Glo microbial cell viability assay kit (Promega, Madison, WI). The luminescence of each sample was detected with the FB12 luminometer (Berthold, Pforzheim, Germany) in three independent experiments. The data were normalized with the CFU count of each sample.

### Detection of membrane potential.

The membrane potential was measured with the BacLight bacterial membrane potential kit (Molecular Probes, Eugene, OR), where the cultures were treated with saline, EDTA, or MgSO_4_. The samples were mixed with 10 μl fluorescent membrane potential indicator dye diethyloxacarbocyanine iodide (DiOC_2_) and incubated at 37°C for another 30 min. The fluorescent signals were recorded by an LSRFortessa flow cytometry analyzer (BD, CA) counting 50,000 cells. Membrane potential was indicated by the ratio between channel F3 (red fluorescence) and F1 (green fluorescence) using FlowJo (BD, CA).

### qRT-PCR.

RNA samples were extracted according to the method previously reported (31). Briefly, the pellets were collected by centrifugation and resuspended in 100 μl diethylpyrocarbonate (DEPC)-H_2_O and 100 μl phenol-chloroform (1:1). The samples were incubated at 70°C for 30 min and then centrifuged. RNA from the supernatants was purified with the RNeasy minikit (Qiagen, Hilden, Germany) according to the protocol provided. Reverse transcription was performed with a cDNA synthesis kit (Bio-Rad, Hercules, CA). The qRT-PCR experiments were carried out with the SYBR green PCR kit (TaKaRa, Japan) on the LightCycler 480 System (Roche, Branchburg, NJ). The primer sequences are listed in Table S3. The data were obtained from three independent experiments, and the threshold cycle (2^−ΔΔ^*^CT^*) method (32) was used for analysis of relative gene expression.

### Statistical analyses.

The signiﬁcance of experimental diﬀerences in persister assay, Mg^2+^ measurement, and intracellular ATP assay was evaluated with the two-tailed unpaired *t* test (two groups).

## References

[1] Balaban NQ, Helaine S, Lewis K, Ackermann M, Aldridge B, Andersson DI, Brynildsen MP, Bumann D, Camilli A, Collins JJ, Dehio C, Fortune S, Ghigo J-M, Hardt W-D, Harms A, Heinemann M, Hung DT, Jenal U, Levin BR, Michiels J, Storz G, Tan M-W, Tenson T, Van Melderen L, Zinkernagel A 2019 Definitions and guidelines for research on antibiotic persistence. Nat Rev Microbiol 17:441–448. doi:10.1038/s41579-019-0207-4. 30980069 PMC7136161

[2] Zhang Y 2014 Persisters, persistent infections and the Yin-Yang model. Emerg Microbes Infect 3:e3. doi:10.1038/emi.2014.3. 26038493 PMC3913823

[3] Conlon BP, Rowe SE, Gandt AB, Nuxoll AS, Donegan NP, Zalis EA, Clair G, Adkins JN, Cheung AL, Lewis K 2016 Persister formation in Staphylococcus aureus is associated with ATP depletion. Nat Microbiol 1:16051. doi:10.1038/nmicrobiol.2016.51. PMC4932909 27572649

[4] Korch SB, Henderson TA, Hill TM 2003 Characterization of the hipA7 allele of Escherichia coli and evidence that high persistence is governed by (p)ppGpp synthesis. Mol Microbiol 50:1199–1213. doi:10.1046/j.1365-2958.2003.03779.x. 14622409

[5] Hansen S, Lewis K, Vulic M 2008 Role of global regulators and nucleotide metabolism in antibiotic tolerance in Escherichia coli. Antimicrob Agents Chemother 52:2718–2726. doi:10.1128/AAC.00144-08. 18519731 PMC2493092

[6] Gollan B, Grabe G, Michaux C, Helaine S 2019 Bacterial persisters and infection: past, present, and progressing. Annu Rev Microbiol 73:359–385. doi:10.1146/annurev-micro-020518-115650. 31500532

[7] Van den Bergh B, Fauvart M, Michiels J 2017 Formation, physiology, ecology, evolution and clinical importance of bacterial persisters. FEMS Microbiol Rev 41:219–251. doi:10.1093/femsre/fux001. 28333307

[8] Fisher RA, Gollan B, Helaine S 2017 Persistent bacterial infections and persister cells. Nat Rev Microbiol 15:453–464. doi:10.1038/nrmicro.2017.42. 28529326

[9] Bernier SP, Lebeaux D, DeFrancesco AS, Valomon A, Soubigou G, Coppée J-Y, Ghigo J-M, Beloin C 2013 Starvation, together with the SOS response, mediates high biofilm-specific tolerance to the fluoroquinolone ofloxacin. PLoS Genet 9:e1003144. doi:10.1371/journal.pgen.1003144. 23300476 PMC3536669

[10] Shan Y, Brown Gandt A, Rowe SE, Deisinger JP, Conlon BP, Lewis K 2017 ATP-dependent persister formation in Escherichia coli. mBio 8:e02267-16. doi:10.1128/mBio.02267-16. 28174313 PMC5296605

[11] Wang Y, Bojer MS, George SE, Wang Z, Jensen PR, Wolz C, Ingmer H 2018 Inactivation of TCA cycle enhances Staphylococcus aureus persister cell formation in stationary phase. Sci Rep 8:10849. doi:10.1038/s41598-018-29123-0. 30022089 PMC6052003

[12] Hussain M, Hastings JG, White PJ 1991 A chemically defined medium for slime production by coagulase-negative staphylococci. J Med Microbiol 34:143–147. doi:10.1099/00222615-34-3-143. 2010904

[13] Li M, Diep BA, Villaruz AE, Braughton KR, Jiang X, DeLeo FR, Chambers HF, Lu Y, Otto M 2009 Evolution of virulence in epidemic community-associated methicillin-resistant Staphylococcus aureus. Proc Natl Acad Sci U S A 106:5883–5888. doi:10.1073/pnas.0900743106. 19293374 PMC2667066

[14] Sassi M, Felden B, Augagneur Y 2014 Draft genome sequence of Staphylococcus aureus subsp. aureus strain HG003, an NCTC8325 derivative. Genome Announc 2:e00855-14. doi:10.1128/genomeA.00855-14. 25169861 PMC4148729

[15] Corsello S, Fulgenzi A, Vietti D, Ferrero ME 2009 The usefulness of chelation therapy for the remission of symptoms caused by previous treatment with mercury-containing pharmaceuticals: a case report. Cases J 2:199. doi:10.1186/1757-1626-2-199. 19946446 PMC2783151

[16] Armitano J, Redder P, Guimarães VA, Linder P 2016 An essential factor for high Mg^2+^ tolerance of Staphylococcus aureus. Front Microbiol 7:1888. doi:10.3389/fmicb.2016.01888. 27933050 PMC5122736

[17] Akanuma G, Kobayashi A, Suzuki S, Kawamura F, Shiwa Y, Watanabe S, Yoshikawa H, Hanai R, Ishizuka M 2014 Defect in the formation of 70S ribosomes caused by lack of ribosomal protein L34 can be suppressed by magnesium. J Bacteriol 196:3820–3830. doi:10.1128/JB.01896-14. 25182490 PMC4248831

[18] Groisman EA, Hollands K, Kriner MA, Lee EJ, Park SY, Pontes MH 2013 Bacterial Mg^2+^ homeostasis, transport, and virulence. Annu Rev Genet 47:625–646. doi:10.1146/annurev-genet-051313-051025. 24079267 PMC4059682

[19] Nierhaus KH 2014 Mg^2+^, K^+^, and the ribosome. J Bacteriol 196:3817–3819. doi:10.1128/JB.02297-14. 25225274 PMC4248827

[20] Pontes MH, Sevostyanova A, Groisman EA 2015 When too much ATP is bad for protein synthesis. J Mol Biol 427:2586–2594. doi:10.1016/j.jmb.2015.06.021. 26150063 PMC4531837

[21] Pontes MH, Yeom J, Groisman EA 2016 Reducing ribosome biosynthesis promotes translation during low Mg^2+^ stress. Mol Cell 64:480–492. doi:10.1016/j.molcel.2016.05.008. 27746019 PMC5500012

[22] Ramesh A, Winkler WC 2010 Magnesium-sensing riboswitches in bacteria. RNA Biol 7:77–83. doi:10.4161/rna.7.1.10490. 20023416

[23] Lee DD, Galera-Laporta L, Bialecka-Fornal M, Moon EC, Shen Z, Briggs SP, Garcia-Ojalvo J, Süel GM 2019 Magnesium flux modulates ribosomes to increase bacterial survival. Cell 177:352–360.e13. doi:10.1016/j.cell.2019.01.042. 30853217 PMC6814349

[24] Beenken KE, Dunman PM, McAleese F, Macapagal D, Murphy E, Projan SJ, Blevins JS, Smeltzer MS 2004 Global gene expression in Staphylococcus aureus biofilms. J Bacteriol 186:4665–4684. doi:10.1128/JB.186.14.4665-4684.2004. 15231800 PMC438561

[25] Fey PD, Endres JL, Yajjala VK, Widhelm TJ, Boissy RJ, Bose JL, Bayles KW 2013 A genetic resource for rapid and comprehensive phenotype screening of nonessential Staphylococcus aureus genes. mBio 4:e00537-12. doi:10.1128/mBio.00537-12. 23404398 PMC3573662

[26] Xu T, Han J, Zhang J, Chen J, Wu N, Zhang W, Zhang Y 2016 Absence of protoheme IX farnesyltransferase CtaB causes virulence attenuation but enhances pigment production and persister survival in MRSA. Front Microbiol 7:1625. doi:10.3389/fmicb.2016.01625. 27822202 PMC5076432

[27] Bae T, Schneewind O 2006 Allelic replacement in Staphylococcus aureus with inducible counter-selection. Plasmid 55:58–63. doi:10.1016/j.plasmid.2005.05.005. 16051359

[28] Xu T, Wu Y, Lin Z, Bertram R, Götz F, Zhang Y, Qu D 2017 Identification of genes controlled by the essential YycFG two-component system reveals a role for biofilm modulation in Staphylococcus epidermidis. Front Microbiol 8:724. doi:10.3389/fmicb.2017.00724. 28491057 PMC5405149

[29] Monk IR, Shah IM, Xu M, Tan M-W, Foster TJ 2012 Transforming the untransformable: application of direct transformation to manipulate genetically Staphylococcus aureus and Staphylococcus epidermidis. mBio 3:e00277-11. doi:10.1128/mBio.00277-11. 22434850 PMC3312211

[30] Maass S, Sievers S, Zühlke D, Kuzinski J, Sappa PK, Muntel J, Hessling B, Bernhardt J, Sietmann R, Völker U, Hecker M, Becher D 2011 Efficient, global-scale quantification of absolute protein amounts by integration of targeted mass spectrometry and two-dimensional gel-based proteomics. Anal Chem 83:2677–2684. doi:10.1021/ac1031836. 21395229

[31] Atshan SS, Shamsudin MN, Lung LT, Ling KH, Sekawi Z, Pei CP, Ghaznavi-Rad E 2012 Improved method for the isolation of RNA from bacteria refractory to disruption, including S. aureus producing biofilm. Gene 494:219–224. doi:10.1016/j.gene.2011.12.010. 22222139

[32] Livak KJ, Schmittgen TD 2001 Analysis of relative gene expression data using real-time quantitative PCR and the 2(-delta delta C(T)) method. Methods 25:402–408. doi:10.1006/meth.2001.1262. 11846609

[33] Chen X, Li G, Liao X, Fang J, Li B, Yu S, Sun M, Wu J, Zhang L, Hu Y, Jiao J, Liu T, Xu L, Chen X, Liu M, Li H, Hu F, Sun K 2019 A switch in the poly(dC)/RmlB complex regulates bacterial persister formation. Nat Commun 10:27. doi:10.1038/s41467-018-07861-z. 30604752 PMC6318315

