# Supplementary material for: Magnesium Links Starvation-Mediated Antibiotic Persistence to ATP
Source: mSphere. 2020 Jan 8;5(1):e00862-19. doi: 10.1128/mSphere.00862-19 (PMC6952205; doi:10.1128/mSphere.00862-19)
Supplement: TABLE S1 [file mSphere.00862-19-st001.docx]

**Supplementary Table 1. Grouping and concentrations of HHWm components.**

| **Group** | **Component** | **Final concentration (mg/L)** |
| --- | --- | --- |
| **Amino acids** | L-aspartic acid | 150 |
|  | L-alanine | 100 |
|  | L-arginine | 100 |
|  | L-cystine | 50 |
|  | Glycine | 100 |
|  | L-glutamic acid | 150 |
|  | L-histidine | 100 |
|  | L-isoleucine | 150 |
|  | L-lysine | 100 |
|  | L-leucine | 150 |
|  | L-methionine | 100 |
|  | L-phenylalanine | 100 |
|  | L-proline | 150 |
|  | L-serine | 100 |
|  | L-threonine | 150 |
|  | L-tryptophan | 100 |
|  | L-tyrosine | 100 |
|  | L-valine | 150 |
| **Glucose** | Glucose | 10,000 |
| **Major salts** | MgSO_4_·7H_2_O | 50 |
|  | Na_2_HPO_4_·2H_2_O | 8,950 |
|  | KH_2_PO_4_ | 3,000 |
|  | (NH_4_)_2_SO_4_ | 2,000 |
|  | CaCl_2_·2H_2_O | 7,50 |
| **Trace salts** | ZnCl_2_ | 1.7 |
|  | MnSO_4_·H_2_O | 3.8 |
|  | FeSO_4_·7H_2_O | 2.8 |
|  | CuCl_2_·2H_2_O | 0.43 |
|  | CoCl_2_·6H_2_O | 0.6 |
|  | Na_2_MoO_4_·2H_2_O | 0.6 |
| **Nucleotides** | Adenine·1/2 H_2_SO_4_ | 3 |
|  | Guanine·1/2 H_2_SO_4_ | 4 |
| **Trace compounds** | Biotin | 0.1 |
|  | Nicotinic acid | 2 |
|  | _D_-Pantothenic acid, Ca salt | 2 |
|  | Pyridoxamine dihydrochloride | 4 |
|  | Riboﬂavin | 2 |
|  | Thiamine hydrochloride | 2 |
