# Supplementary material for: Magnesium Links Starvation-Mediated Antibiotic Persistence to ATP
Source: mSphere. 2020 Jan 8;5(1):e00862-19. doi: 10.1128/mSphere.00862-19 (PMC6952205; doi:10.1128/mSphere.00862-19)
Supplement: TABLE S2 [file mSphere.00862-19-st002.docx]

**Supplementary Table 2. Strains and plasmids used in this study.**

| **Strain/plasmid** | **Specification** | **Reference** |
| --- | --- | --- |
| **Strains** | | |
| DC10B | A DC10B derivate for plasmid amplification | (1) |
| Newman | An MSSA strain from a clinical sample | (2) |
| ΔyhdE | The *yhdE* mutant of Newman | This study |
| USA300 LAC |  | (3) |
| ΔsrrB | The *srrB* mutant of USA300 | (4) |
| ΔphoR | The *phoR* mutant of USA300 | (4) |
| USA500 |  | (5) |
| ΔarlRS | The *arlRS* mutant of USA500 | (6) |
| ΔgraRS | The *graRS* mutant of USA500 | (6) |
| **Plasmids** | | |
| pMX6 | ATc inducible antisense RNA expressing plasmid, Cm^R^, Amp^R^ | (7) |
| pMXmgtE | The asRNA plasmid for gene knockdown of *mgtE* | This study |
| pMXwalR | The as RNA plasmid for gene knock down of *walR* | This study |

Cm^R^, chloramphenicol resistance; Amp^R^, ampicillin resistance.

**References:**

1. Monk IR, Shah IM, Xu M, Tan MW, Foster TJ. 2012. Transforming the untransformable: application of direct transformation to manipulate genetically Staphylococcus aureus and Staphylococcus epidermidis. MBio 3.

2. Baba T, Bae T, Schneewind O, Takeuchi F, Hiramatsu K. 2008. Genome sequence of Staphylococcus aureus strain Newman and comparative analysis of staphylococcal genomes: polymorphism and evolution of two major pathogenicity islands. J Bacteriol 190:300-10.

3. Voyich JM, Braughton KR, Sturdevant DE, Whitney AR, Said-Salim B, Porcella SF, Long RD, Dorward DW, Gardner DJ, Kreiswirth BN, Musser JM, DeLeo FR. 2005. Insights into mechanisms used by Staphylococcus aureus to avoid destruction by human neutrophils. J Immunol 175:3907-19.

4. Fey PD, Endres JL, Yajjala VK, Widhelm TJ, Boissy RJ, Bose JL, Bayles KW. 2013. A genetic resource for rapid and comprehensive phenotype screening of nonessential Staphylococcus aureus genes. MBio 4:e00537-12.

5. Li M, Diep BA, Villaruz AE, Braughton KR, Jiang X, DeLeo FR, Chambers HF, Lu Y, Otto M. 2009. Evolution of virulence in epidemic community-associated methicillin-resistant Staphylococcus aureus. Proceedings of the National Academy of Sciences 106:5883.

6. Xu T, Wang X-Y, Cui P, Zhang Y-M, Zhang W-H, Zhang Y. 2017. The Agr Quorum Sensing System Represses Persister Formation through Regulation of Phenol Soluble Modulins in Staphylococcus aureus. Frontiers in Microbiology 8.

7. Xu T, Wu Y, Lin Z, Bertram R, Götz F, Zhang Y, Qu D. 2017. Identification of genes controlled by the essential YycFG two-component system reveals a role for biofilm modulation in Staphylococcus epidermidis. Frontiers in Microbiology 8.
