# Supplementary material for: Magnesium Links Starvation-Mediated Antibiotic Persistence to ATP
Source: mSphere. 2020 Jan 8;5(1):e00862-19. doi: 10.1128/mSphere.00862-19 (PMC6952205; doi:10.1128/mSphere.00862-19)
Supplement: TABLE S3 [file mSphere.00862-19-st003.docx]

**Supplementary Table 3. Primers used in this study.**

| **Primer** | **Sequence (5’→3’)** |
| --- | --- |
| **For construction of antisense RNA plasmids** | |
| asrmgtE-f | GAAGATCTATGTCAATGAACACAGATGAAAAAG |
| asrmgtE-r | AGCGGCCGCATATGTGTGTAATGCTAGAAATTCAT |
| asryycF-f | gaagatctGATTTAAGAAAAGAGGTTTATGCAAAT |
| asryycF-r | agcggccgCATCATTACCATCGTATGCACA |
| **For construction of knockout strains** | |
| yhdp-uf-KpnI | GGGGTACCTCGACTGATAAAATAGATAATGCACTT |
| yhdp-ur | CATTCTTAGTTGGTTTATATATTAATATTTAATTCCCTATTTCTAGGGATTC |
| yhdp-df | GAATCCCTAGAAATAGGGAATTAAATATTAATATATAAACCAACTAAGAATG |
| yhdp-dr-MluI | CGACGCGTATTAAAACAAGGAATGACGACTCTAAT |
| **For qRT-PCR** | |
| RTcap5A-f | TATCATCGCTGGCATCATTA |
| RTcap5A-r | AGACCTCCTTTGTTTGTTATG |
| RTarcA-f | GTGATGTCATTGATGGTGCTA |
| RTarcA-r | ACTACAACGCCTGGTCTTA |
| RTmgtE-f | GTGTTCAAGAGGAATTATACGA |
| RTmgtE-r | GCAACTTCTTCAGGTGATAA |
| RTyhdP-f | ATAGGTGGATGGTTACAATCT |
| RTyhdP-r | GATAGTAGGTCTCGCTTCAT |
| RTrrs1-f | GTGCTACAATGGACAATACAA |
| RTrrs1-r | ACTACAATCCGAACTGAGAA |
